# Intradermal Application of Crotamine Induces Inflammatory and Immunological Changes In Vivo

**DOI:** 10.3390/toxins11010039

**Published:** 2019-01-14

**Authors:** Ana Vitória Pupo Silvestrini, Luana Henrique de Macedo, Thiago Antônio Moretti de Andrade, Maíra Felonato Mendes, Acácio Antônio Pigoso, Maurício Ventura Mazzi

**Affiliations:** 1 School of Pharmaceutical Sciences of Ribeirão Preto, University of São Paulo, Av. do Café, s/n, CEP 14040-903 Ribeirão Preto, SP, Brazil; anapupo@usp.br (A.V.P.S.); luana.hdm@usp.br (L.H.d.M.); 2 Graduate Program in Biomedical Sciences Hermínio Ometto University Center, FHO-UNIARARAS, Av. Dr. Maximiliano Baruto, 500, CEP 13607-339 Araras, SP, Brazil; thiago.andrade@fho.edu.br (T.A.M.d.A.); mairafelonato@fho.edu.br (M.F.M.); acaciopigoso@uniararas.br (A.A.P.)

**Keywords:** *Crotalus durissus terrificus*, crotamine, cell-penetrating peptide, inflammation

## Abstract

Crotamine is a single-chain polypeptide with cell-penetrating properties, which is considered a promising molecule for clinical use. Nevertheless, its biosafety data are still scarce. Herein, we assessed the in vivo proinflammatory properties of crotamine, including its local effect and systemic serum parameters. Sixty male *Wistar* rats were intradermically injected with 200, 400 and 800 µg crotamine and analyzed after 1, 3 and 7 days. Local effect of crotamine was assessed by determination of MPO and NAG activities, NO levels and angiogenesis. Systemic inflammatory response was assessed by determination of IL-10, TNF-α, CRP, NO, TBARS and SH groups. Crotamine induced macrophages and neutrophils chemotaxis as evidenced by the upregulation of both NAG (0.5–0.6 OD/mg) and MPO (0.1–0.2 OD/mg) activities, on the first and third day of analysis, respectively. High levels of NO were observed for all concentrations and time-points. Moreover, 800 μg crotamine resulted in serum NO (64.7 μM) and local tissue NO (58.5 μM) levels higher or equivalent to those recorded for their respective histamine controls (55.7 μM and 59.0 μM). Crotamine also induced a significant angiogenic response compared to histamine. Systemically, crotamine induced a progressive increase in serum CRP levels up to the third day of analysis (22.4–45.8 mg/mL), which was significantly greater than control values. Crotamine (400 μg) also caused an increase in serum TNF-α, in the first day of analysis (1095.4 pg/mL), however a significant increase in IL-10 (122.2 pg/mL) was also recorded for the same time-point, suggesting the induction of an anti-inflammatory effect. Finally, crotamine changed the systemic redox state by inducing gradual increase in serum levels of TBARS (1.0–1.8 μM/mL) and decrease in SH levels (124.7–19.5 μM/mL) throughout the experimental period of analysis. In summary, rats intradermally injected with crotamine presented local and systemic acute inflammatory responses similarly to histamine, which limits crotamine therapeutic use on its original form.

## 1. Introduction

Crotamine is a basic polypeptide toxin found in the venom of the rattlesnake *Crotalus durissus terrificus* and a member of the α-myotoxin family. Its three-dimensional structure αβ1β2β3 is similar to that of other human proteins intrinsically related to antimicrobial activity, such as β-defensins. Furthermore, positively charged regions distributed throughout the structure and a small area of negative charge optimize electrostatic interactions between crotamine and diverse cell membranes [1,2,3,4,5].

This toxin displays different cellular and molecular targets as well as several activities, including neurotoxicity and myotoxicity. Its myotoxic potential is related to the electrophysiological changes in sodium and potassium channels, changes in mitochondrial calcium homeostasis and degeneration of myofibrils, with consequent structural damage to muscle fibers [6,7,8,9]. Moreover, studies have shown that the mechanism of action of crotamine is not restricted to the muscle tissue, involving other tissues, mainly liver and kidneys or involving other cells such as fibroblasts, neural and embryonic stem cells [2,10].

In addition to its toxic effect, crotamine has been shown to potentiate insulin release [11] and to have a strong antimicrobial activity [9,12,13,14,15]. Other properties, still poorly understood, include analgesic and hemolytic activities, as well as stimulation of the immune system by interfering with the activity of mast cells, macrophages, lymphocytes and monocytes. [12,16,17,18].

Crotamine also has cell-penetrating ability and nuclear specificity, acting through independent mechanisms of energy expenditure from interaction with extracellular matrix proteoglycans [10,19]. Therefore, crotamine has been studied as a nucleolar targeting peptide (NrTP) for biomolecules and antitumor agents on different tumoral strains [3,17,20,21]. The cytotoxic effects of crotamine have been demonstrated in vivo and in vitro using tumor cell lines, allowing the study of the mechanisms by which the molecule can alter cellular homeostasis by inducing damage to cytoplasmic organelles such as lysosomes and mitochondria [19,22].

Due to its pharmacological potential, crotamine is considered a promising molecule for clinical use in different biomedical fields [2,3,23]. However, data on its local and systemic safety in biological models are scarce [16,24,25]. Thus, to better understand the in vivo proinflammatory activity of crotamine, we assessed the effects of this toxin on different immunological parameters.

## 2. Results

### 2.1. Crotamine Induced C-Reactive Protein Production

**Figure 1:**
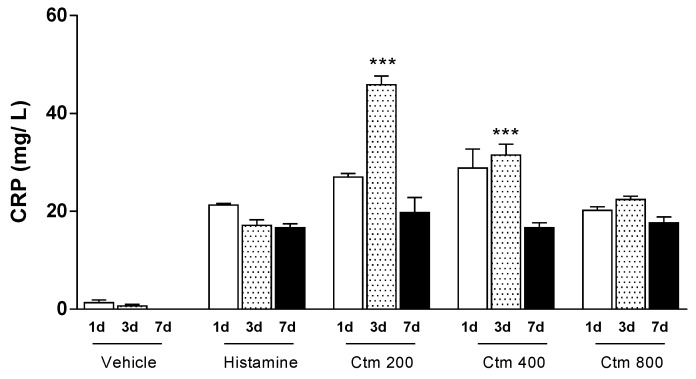
Serum levels of C-reactive protein (CRP). Effect of Crotamine (200, 400 and 800 μg Ctm) intradermal injection after 1, 3 and 7 days on serum CRP levels, determined spectrophotometrically (λ_540nm_). Positive control: 1000 μg histamine. Negative control: sterile H_2_O (vehicle). Results are reported as mean ± standard deviation. As appropriate, *p* < 0.05 was set as significance level. *** *p* < 0.001 in relation to the positive control and between groups (*n* = 4 animals/analysis/time). Comparison between data was done by one-way ANOVA and Tukey post-test using GraphPad Prism 6.0 software (GraphPad Software, Inc., San Diego, CA, USA).

C-reactive protein (CRP) is an important marker of acute inflammation in response to various stimuli caused by infectious agents or tissue damage and it was thus used to evaluate the inflammatory effect of crotamine. A progressive increase in serum CRP levels was observed up to the third day of analysis for all groups treated with crotamine. However, the highest average serum CRP level recorded was 45.8 mg/L after treatment with 200 μg crotamine, while an average CRP level of 31.5 mg/L was recorded after treatment with 400 μg crotamine, on the third day of analysis (Figure 1). All doses of crotamine resulted in average serum levels of CRP equal to or greater than those induced by 1000 μg histamine on the respective days of analysis. Unlike histamine treatment, which induced a sudden peak of CRP equal to 21.3 mg/L on the first day followed by a gradual decrease in serum CRP levels up to the seventh day, administration of 200 µg crotamine increased serum CRP levels by the third day (45.8 mg/L) and decreased thereafter until the seventh day (22.4 mg/L). In addition, crotamine had a more intense and more prolonged effect than histamine but this effect decreased with the highest dose.

### 2.2. Crotamine Affected Pro- and Anti-Inflammatory Systemic Cytokines

**Figure 2:**
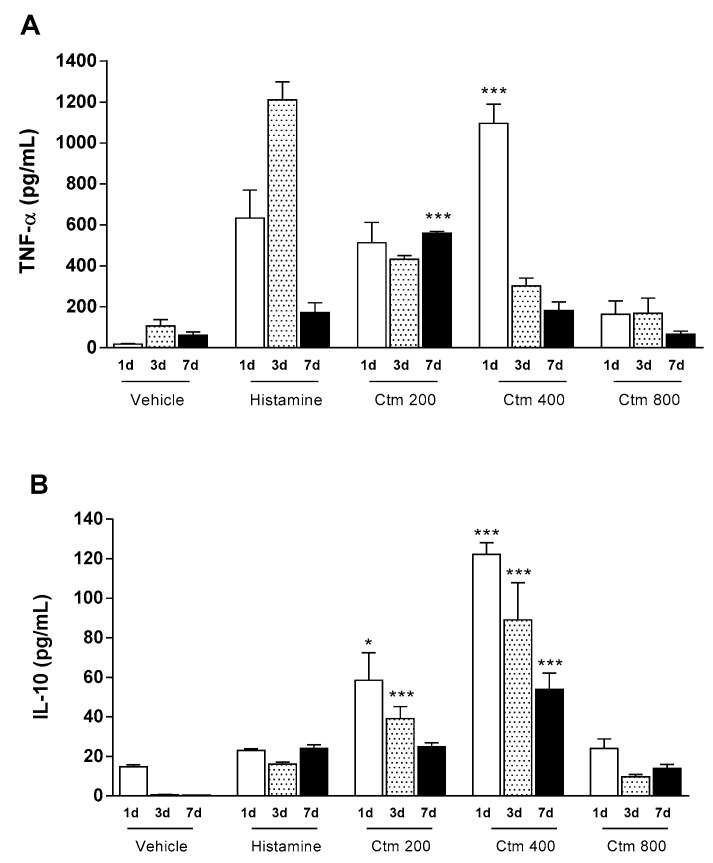
Serum levels of TNF-α and IL-10. Effect of intradermal injection of crotamine (200, 400 and 800 μg Ctm) after 1, 3 and 7 days on (**A**) serum TNF-α, determined by ELISA (λ_540nm_); and (**B**) serum IL-10, determined by ELISA (λ_540nm_). Positive control: 1000 μg histamine. Negative control: sterile H_2_O (vehicle). Results are reported as mean ± standard deviation. As appropriate, *p* < 0.05 was set as significance level. * *p* < 0.05 and *** *p* < 0.001 in relation to the positive control and between groups (*n* = 4 animals/analysis/time). Comparison between data was done by one-way ANOVA and Tukey post-test using GraphPad Prism 6.0 software (GraphPad Software, Inc., San Diego, CA, USA).

In response to an aggressive stimulus there is a release of inflammatory mediators (products from activated leukocyte and platelets, arachidonic acid metabolism, coagulation and complement cascades) that act locally or systemically depending on the intensity and duration of the injury. Such mediators are soluble molecules derived from cells, plasma or matrix proteins that act on target inflammatory cells through specific receptors and are responsible for vascular and cellular responses, both in acute and chronic stimuli. To study this effect, cytokines TNF-α and IL-10 were determined in animals treated with crotamine and compared to the control groups. The results presented in Figure 2A show that there was an increase in the levels of serum TNF-α in animals treated with doses of crotamine ranging from 200 to 800 μg, followed by a reduction at a later stage. However, animals treated with 400 μg crotamine showed a significantly greater level of serum TNF-α after the first day of treatment (1095.4 pg/mL), when compared to the level of serum TNF-α in animals treated with 1000 μg histamine (633.4 pg/mL). The results also showed a significant increase in IL-10 serum levels within the first three days of analysis (ranging from 58.5 to 122.2 pg/mL), in animals treated with 200 and 400 μg crotamine, followed by a decrease in serum concentration of IL-10 on the seventh day of assessment (Figure 2B). Interestingly, the highest dose of crotamine (800 μg) resulted in lower serum levels of TNF-α and IL-10 (163.1 and 24.0 pg/mL, respectively), on the first day after crotamine administration. In contrast, histamine treatment induced the highest level of TNF-α on the third day of assessment (1211.2 pg/mL) and a lower level of serum IL-10 (23.0 pg/mL), when compared to groups treated with different concentrations of crotamine.

### 2.3. Crotamine Induces Increased Levels of Nitric Oxide and Cellular Markers

**Figure 3:**
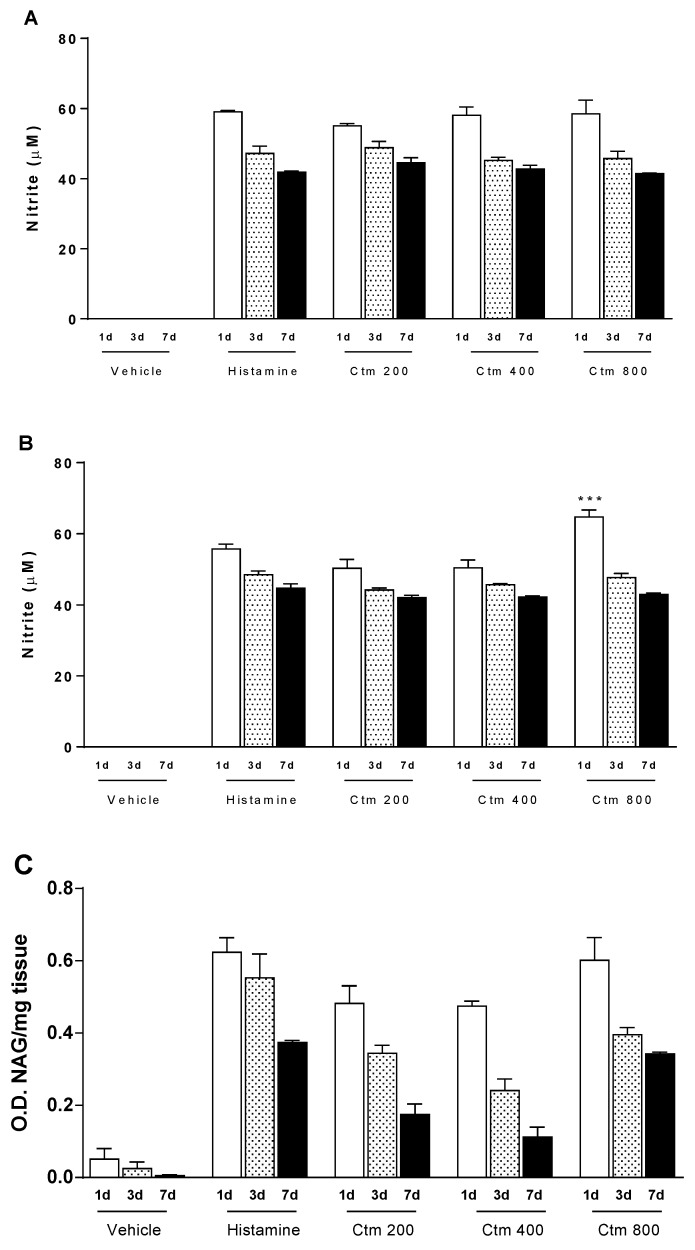

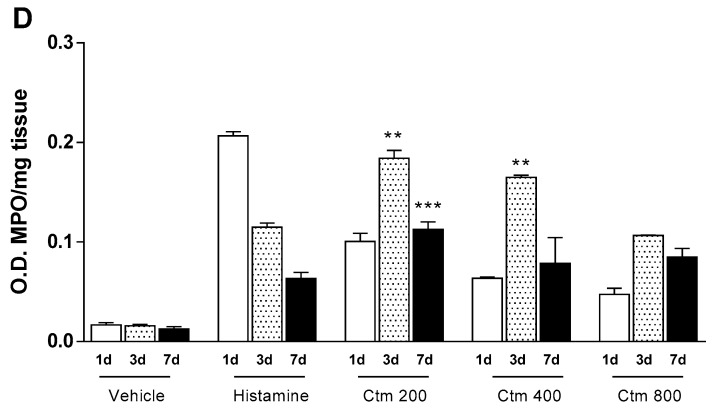
Serum and tissue levels of NO and cellular markers. Effect of intradermal injection of crotamine (200, 400 and 800 μg Ctm) after 1, 3 and 7 days on nitric oxide in (**A**) previously homogenized tissue and (**B**) serum, determined by the Griess reaction (λ_570nm_). (**C**) NAG: N-Acetylglicosaminidase (macrophage activity marker); (**D**) MPO: myeloperoxidase (neutrophil activity marker). Positive control: 1000 μg histamine. Negative control: sterile H_2_O (vehicle). Results are reported as mean ± standard deviation. As appropriate, *p* < 0.05 was set as significance level. ** *p* < 0.01 and *** *p* < 0.001 in relation to the positive control and between groups (*n* = 4 animals/analysis/time). Comparison between data was done by one-way ANOVA and Tukey post-test using GraphPad Prism 6.0 software (GraphPad Software, Inc., San Diego, CA, USA).

In the immune system, high levels of NO inhibit neutrophil functions, thus, displaying an anti-inflammatory effect. N-acetylglucosaminidase (NAG) and myeloperoxidase (MPO) are biomarkers of macrophage and neutrophil activity, respectively. In this study, crotamine and histamine significantly induced NO production when compared with the negative control. The increase of NO levels in both homogenized tissue (Figure 3A) and serum (Figure 3B) was achieved at all crotamine and histamine concentrations tested, during the seven days of analysis. Treatment with 800 μg crotamine resulted in serum NO (64.7 μM) and local tissue NO (58.5 μM) levels higher or equivalent to those recorded for their respective histamine controls (55.7 μM serum NO and 59.0 μM tissue NO), as observed in Figure 3A,B. In addition, doses of crotamine ranging from 200 to 800 μg induced upregulation of NAG activity in tissue samples (0.5 to 0.6 OD/mg tissue) on the first day of analysis, followed by a gradual decrease in enzyme activity up to the seventh day. Conversely, the lowest concentration of crotamine (200 μg), induced the highest MPO activity (0.2 OD/mg tissue), while the highest concentration of crotamine (800 μg) had the opposite trend (0.1 OD/mg tissue) on the third day of analysis (Figure 3D). MPO elevation in tissues corroborates neutrophil infiltration observed in tissue sections from the injured dorsal skin, by light field microscopy (Figure S1).

### 2.4. Crotamine Changed the Systemic Redox State

**Figure 4:**
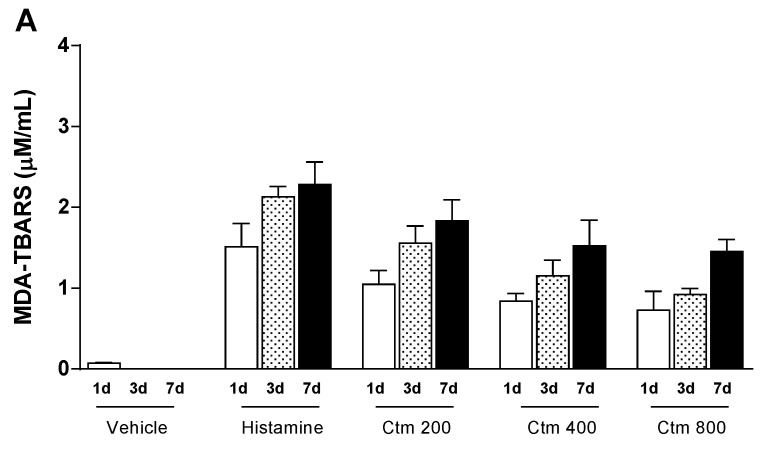

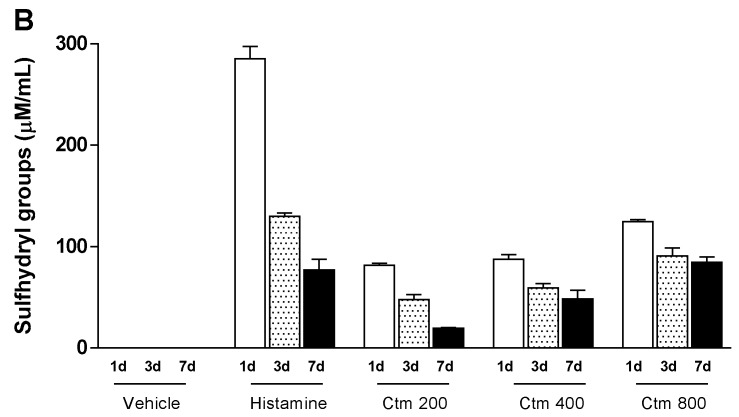
Serum levels of oxidative stress markers. Effect of intradermal injection of crotamine (200, 400 and 800 μg Ctm) on redox state after 1, 3 and 7 days. (**A**) Analysis of thiobarbituric acid reactive substances (TBARS) in serum by spectrophotometric determination (λ_532nm_). (**B**) Analysis of sulfhydryl groups (SH) in serum by spectrophotometric determination (λ_412nm_). Positive control: 1000 μg histamine. Negative control: sterile H_2_O (vehicle). Results are reported as mean ± standard deviation. As appropriate, *p* < 0.05 was set as significance level in relation to the positive control and between groups (*n* = 4 animals/analysis/time). Comparison between data was done by one-way ANOVA and Tukey post-test using GraphPad Prism 6.0 software (GraphPad Software, Inc., San Diego, CA, USA).

In order to understand the relationship between crotamine-induced inflammatory process and oxidative stress, serum levels of lipid peroxidation products (MDA-TBARS) and sulfhydryl groups (-SH) were determined. The serum levels of TBARS in animals injected intradermally with either crotamine (200 to 800 μg) or histamine (1000 μg) gradually increased, suggesting that the stress trigger remained active throughout the experimental period of analysis. The mean serum levels of TBARS in the experimental groups of animals treated with 200, 400 and 800 μg crotamine increased from 1.0 to 1.8 μM/mL, 0.8 to 1.5 μM/mL and 0.7 to 1.5 μM/mL, respectively, while 1000 μg histamine induced a rise in TBARS levels from 1.5 to 2.3 μM/mL over a seven-day period of analysis (Figure 4A). On the other hand, the mean serum levels of sulfhydryl groups in the experimental groups of animals treated with 200, 400 and 800 μg crotamine gradually decreased from 81.8 to 19.5 μM/mL, 87.6 to 48.6 μM/mL and 124.7 to 84.5 μM/mL, respectively, while 1000 μg histamine induced a drop in sulfhydryl groups levels from 285.2 to 77.2 μM/mL over the same period of analysis (Figure 4B). Although crotamine was able to alter oxidative stress parameters significantly, the pro-oxidant effect of this toxin was lower than that recorded for histamine, in the established experimental conditions.

### 2.5. Crotamine Stimulated Angiogenesis

**Figure 5:**
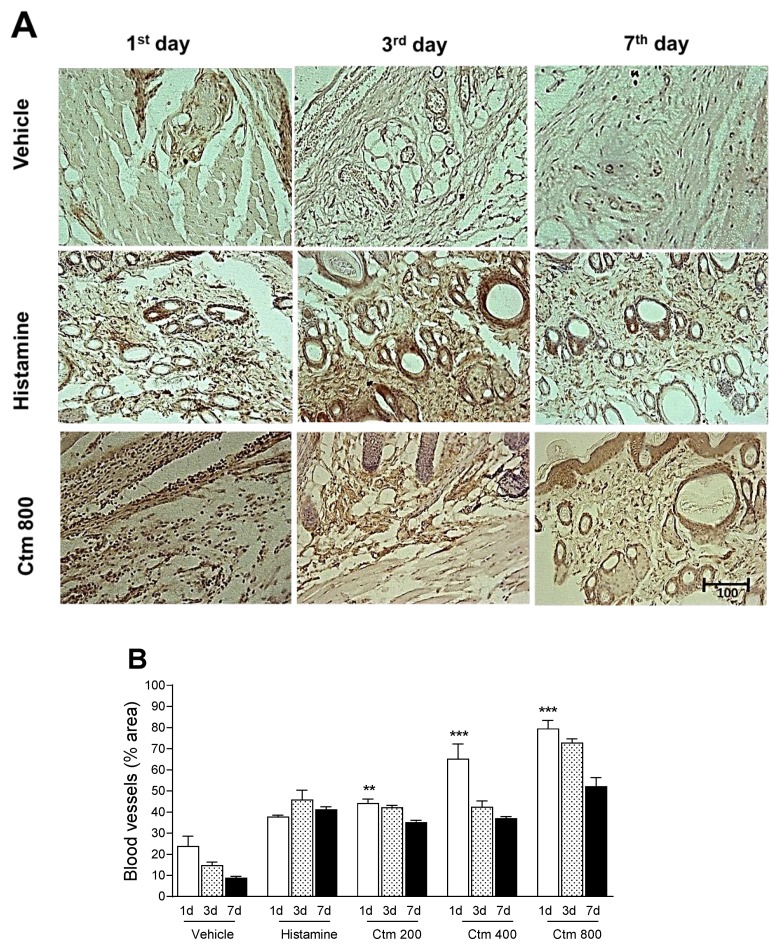
Crotamine-induced local angiogenesis. (**A**) Photomicrographies of cross-sections from the dorsal skin (deep dermis) of Wistar rats 1, 3 and 7 days after intradermal injections of 800 μg crotamine: newly formed blood vessels were evidenced by immunohistochemical analysis of VEGF expression (final magnification, ×200). (**B**) Vascular density analysis of treatment and control groups. Treatment: crotamine (200, 400 and 800 μg Ctm); Positive control: 1000 μg histamine; Negative control: sterile H_2_O (vehicle). The Plugin "Color Deconvolution,” by ImageJ 1.48 v software (National Institutes of Health, Bethesda, MD, USA) was used for VEGF quantification. The sections were counterstained with Harris’s hematoxylin method and analyzed by light field microscopy. Bar = 100 μm. Results are reported as mean ± standard deviation. As appropriate, *p* < 0.05 was set as significance level. ** *p* < 0.01 and *** *p* < 0.001 in relation to the positive control and between groups (*n* = 4 animals/analysis/time). Comparison between data was done by one-way ANOVA and Tukey post-test using GraphPad Prism 6.0 software (GraphPad Software, Inc., San Diego, CA, USA).

Figure 5A shows representative photomicrographic images of cross-sections obtained from the dorsal skin of animals euthanized 1, 3 and 7 days after intradermal injections of 800 μg crotamine, 1000 μg histamine (positive control) and sterile water as vehicle (negative control). Crotamine-induced angiogenesis and VEGF expression were measured by morphometric and immunohistochemical analysis of skin biopsies from treated animals, 1, 3 and 7 days post intradermal injection of 200, 400 and 800 μg toxin and compared with data from control groups, as shown in Figure 5B. Crotamine (200, 400 and 800 μg) significantly induced upregulation of VEGF, 24 h after intradermal injection when compared to its respective negative control. However, this event was followed by downregulation of VEGF between the 3rd and 7th day of analysis, as the blood vessels density was lowered, although values for crotamine-treated groups were still greater than or equal to those recorded for the control groups.

## 3. Discussion

Snake venoms contain a diversity of biologically active polypeptides with remarkable applications in biotechnology due to their ability to cause physiological and biochemical changes on different biological systems [26]. In addition to their use in cancer cells, cardiovascular system and in neurochemistry, advances have been made on the composition and toxicity of poisons for the development of antivenoms [27,28,29,30,31]. Thus, a number of toxins and peptides isolated to date has been found to interact with ion channels, enzymes and components of the cell membrane [26,29,32], resulting in activities such as analgesic [26], antimicrobial [33,34,35], antihypertensive [32], antiviral [36], antiparasitic [37,38] and antitumor [39,40,41,42,43].

The pharmacological properties of crotamine on different biological models include its analgesic potential [16], insulin release potential [11], memory persistence enhancement without psychomotor alterations [44] and antimicrobial and antiparasitic actions in several species [5,12,13,14,15,23]. Moreover, due to its cell-penetrating capacity and site-specific interactions, crotamine has been studied as a drug-mediating peptide and as a model for the discovery of new antitumor molecules [10,17,19,21,22,23]. However, data on crotamine safety for local and systemic application in biological models are scarce [24,25].

In this study, the intradermal (*id*) route of administration was evaluated, considering the ability of crotamine to penetrate cells and also considering the presence of many lymphatic structures in the dermis, which contribute to the drainage and diffusion of molecules [45]. We demonstrated that a single *id* injection of crotamine, at all studied concentrations and time-points, induced acute changes in immunological parameters and promoted oxidative stress but did not present a dose- or time-dependent response.

Although assessed separately, the metabolic and immune pathways are interdependent, since hormones, cytokines, transcription factors and signaling proteins act in both pathways to maintain the body’s homeostasis. The innate and the adaptive immune systems have numerous components with protective functions [46]. The inflammatory response, on the other hand, is initiated by inflammatory cytokines such as IL-1, TNF-α and IL-6 released from an affected tissue inducing hepatic synthesis of acute phase proteins, such as CRP [47].

In this study, crotamine increased CRP levels on the third day after intradermal injection (Figure 1). CRP is an acute inflammation signaling protein, mainly produced by the liver but also by adipocytes and arterial tissue and regulated by cytokines IL-6, TNF-α and IL-1 [48]. CRP levels are increased in response to active infections and acute inflammatory processes [48]. Thus, our results suggest that crotamine stimulates the production of CRP either in response to the local aggression or via TNF-α signaling, whose levels were also shown to be high (Figure 2A). 

The elevated TNF-α level on the first day of analysis after intradermal injection of crotamine indicates its potential effect on an initial inflammatory response and the increase of other immunological markers involved in the process. TNF-α is mainly responsible for the recruitment and activation of neutrophils and monocytes in the injury site. In low concentrations, it acts on endothelial cells promoting vasodilation and stimulating the secretion of other chemotactic cytokines and fibroblasts [49,50,51]. 

In contrast, our results showed that crotamine might exert two opposite effects on the synthesis and signaling of cytokines in inflammatory and immunological processes. Animals injected with lower crotamine doses (200 and 400 μg) showed significantly increased levels of IL-10 when compared to histamine control group (Figure 2B). IL-10 has an anti-inflammatory action by regulating the activity and production of pro-inflammatory cytokines by macrophages, monocytes, mast cells and dendritic cells, thus limiting the inflammatory and immunological response [46,49]. 

Levels of proinflammatory cytokines, such as IL-6, were correlated with the myotoxic and edematogenic effects of crotamine isoforms isolated from *C. d. cumumensis* in the study by Ponce-Soto et al. [52]. Studies on the proinflammatory effects of crotamine demonstrated that this peptide increased the phagocytic activity in macrophages associated with NO, TNF-α and IL-1β, which are strictly related to inflammatory responses [18]. 

The cell response to stimuli depends on a complex signaling process. The stimuli are transmitted from the extracellular medium to the intracellular medium through an orderly sequence of reactions, some of them dependent on oxidation reactions, generally referred to as redox-sensitive signaling [53]. Thus, along with the analysis of cytokines and signaling molecules that mediate an inflammatory response, oxidative stress biomarkers can provide information on the relationship between oxidative damage, macromolecules (DNA, lipids and proteins) and various inflammatory and immunological processes [54].

Herein, crotamine was shown to induce systemic oxidative stress, which was evidenced by the higher serum levels of MDA-TBARS, when compared with the histamine control group (Figure 4B). However, this effect diminished with increasing doses of crotamine, suggesting a positive correlation between crotamine-induced pro-oxidant profile and the high serum levels of MPO (Figure 3D), since increased levels of MPO activity represent an important marker of inflammation and oxidative stress [51]. On the other hand, our results suggest that the highest concentration of crotamine (800 μg) stimulated the anti-oxidant system in order to reduce the oxidative damage, as indicated by the high serum levels of -SH groups (Figure 4B). Consequently, TNF-α and IL-10 levels appear to peak at medium dose and then decrease with a higher concentration of crotamine (Figure 2), suggesting an anti-inflammatory activity of this toxin.

Regarding to the NAG activity, an opposite trend was observed, as presented in Figure 3C. Upregulation of NAG was observed on the first day of analysis demonstrating that anti-inflammatory activity of crotamine was not persistent. Additionally, an expressive increase of NO levels was recorded along the seven days of analysis, at all crotamine concentrations tested (Figure 3A,B). NO is a potentially toxic agent, whose toxicity could be particularly denoted in stress oxidative conditions. Thus, our results suggest that NO may play a key role in the activity of macrophages [55,56,57]. 

Using an intra-hippocampal application route, Gonçalves et al. [25] observed that crotamine altered the oxidative parameters in the serum of animals after 21 days. These results suggest that crotamine has the ability to induce an imbalance in the systemic redox system for a long time, regardless of the route of administration. Previous studies on *Crotalus durissus terrificus* venom and its main component, crotoxin, have shown that NO is closely related to antinociceptive effect [58], modulation of macrocytic activity [41,59,60] and the myotoxic effect of the venom [61]. 

Defensins and cathelicidins are the best characterized antimicrobial peptides which act as effectors of the innate immune response [62]. In addition, several antimicrobial peptides appear to initiate the process of tissue repair, mainly by inducing an angiogenic response at the site of injury [63]. Crotamine has a heterogeneous cytotoxic profile on different microorganisms, as well as structural and/or genetic similarities with antimicrobial peptides such as β-defensins [64].

Morphometric analysis of immunohistochemistry data presented in Figure 5A,B showed an upregulation of VEGF 24 h after the intradermal injection of crotamine. It was also observed that between the 3rd and 7th day of analysis there was a downregulation of the number of newly formed blood vessels. These values, however, were still greater than or equal to those determined for the histamine-treated control group.

To date, little is known about the immunomodulatory and immunogenic properties of venom components. Cell-penetrating peptides (CPPs) have attracted considerable attention as a new class of ligands for delivery of specific therapeutic and diagnostic agents mainly due to several advantages compared to conventional antibodies, such as easier synthesis, smaller sizes, lower immunogenicity and cytotoxicity, besides offering simpler and improved conjugation to nanocarriers. Immunogenicity is the main cause of failure of biological products in clinical trials and therefore it is imperative that drug development studies include an immunogenicity risk assessment, leading to a clinical strategy [64,65,66,67,68].

## 4. Conclusions

Although muscle necrosis is the most significant medical problem associated with small myotoxins, our observations suggest that the biological significance of these molecules is associated to their ability to cause local and systemic damage, depending on their pharmacokinetic properties. In view of the above, we conclude that the intradermal injection of crotamine in rats induced local and systemic acute inflammatory responses that limit the therapeutic use of this peptide in its original form. In addition, we demonstrated that this toxin has a similar effect to histamine in vivo.

## 5. Materials and Methods

### 5.1. Materials and Venom

The yellowish venom of *Crotalus durissus terrificus* was purchased from Koemitã serpentary (Mococa, SP, Brazil). All other reagents used in this study were of analytical grade and purchased from BD Bioscience^®^ (San Jose, CA, USA), Sigma Chem. Co^®^ (St. Louis, MO, USA), Merck^®^ (Darmstadt, Germany), Leica^®^ (Wetzlar, Germany) and Sinapse Biotechnologia^®^ (São Paulo, SP, Brazil).

### 5.2. Purification of Crotamine

Crotamine was isolated from *Crotalus durissus terrificus* venom by using Heparin-Sepharose affinity chromatography. Heparin-Sepharose FF (HiTrap, heparin (HP), 5 mL) column was previously equilibrated with 0.01M sodium phosphate, pH 7.0. The protein was eluted by a NaCl gradient (0–1.5 M) at a flow rate of 2.5 mL/min and 3 mL fractions were collected. Next, crotamine was subjected to functional characterization. All purification and identification procedure of crotamine were performed according to the methodology previously described by Batista da Silva et al. [9].

### 5.3. Animals

Sixty male Wistar rats (*Rattus norvegicus albinus*) of 250 ± 50 g were used in this study. Animals were housed in polycarbonate cages under constant temperature (23 °C) and humidity (55%), with a 12 h light/dark cycle and access to food and water *ad libitum*. All procedures were approved by the Animal Ethics Committee of the Institution (protocol n° 058/2016), which follows the guidelines of the Brazilian College of Animal Experimentation (COBEA) as well as Brazilian law for scientific use of animals (Law n°. 11,794, of 8 October 2008).

### 5.4. Experimental Design and Sample Collection

Animals were randomly divided into five groups of twelve as follows: negative control group (injected intradermally with vehicle (sterile water)), positive control group (injected intradermally with 1000 μg histamine) and three crotamine-treated groups (injected intradermally with sublethal doses of 200, 400 and 800 μg crotamine). Each experimental group received a single intradermal injection (100 μL) according to the good practice guide for substance administration [69]. After 1, 3 and 7 days, the animals were anesthetized by IM administration of xylazine hydrochloride/ketamine hydrochloride solution, tissue samples were collected from the injection site and blood was collected by cardiac puncture at each time-point. Samples were stored at −80 °C for further analysis. Inflammatory and immunological parameters were assessed by immunoenzymatic assays for Interleukin-10 (IL-10) and tumor necrosis factor-α (TNF-α) or by biochemical assays for C-reactive protein (CRP), nitric oxide and redox level. Tissue samples were subjected to biochemical analysis for myeloperoxidase, N-acetylglucosaminidase and nitric oxide activities.

### 5.5. Clinical Parameters for Immunological and Inflammatory Evaluation

#### 5.5.1. C-Reactive protein (CRP) and Cytokines Measurements

A 5-mL blood sample was collected from each animal by cardiac puncture. Serum was obtained by centrifugation at 1000× *g* for 10 min, aliquoted and then frozen at −80 °C. Serum IL-10 and TNF-α levels were measured by Enzyme-Linked Immunosorbent Assay (ELISA) using commercially available monoclonal antibodies and biotinylated polyclonal antibodies (BD Bioscience^®^), according to the manufacturer’s recommendations. The absorbance was measured spectrophotometrically (λ_540nm_) in an automatic microplate reader system ELx 800 (BioTek Instruments, Inc., Winooski, VT, USA). Assays were performed in triplicate for each parameter assessed. Concentration levels of IL-10 and TNFα cytokines were presented in pg/mL of serum with a detection sensitivity limit of 1 pg/mL.

Serum CRP levels were measured using the reagent kit Turbiquest^®^ Plus CRP (Labtest Diagnóstica S.A., Lagoa Santa, MG, Brazil), according to the manufacturer’s recommendations. Presence of CRP in the test specimen results in the formation of an insoluble complex producing a turbidity, which is measured at 540 nm. Data were calculated as the absorbance change per minute (1 and 5 min) and reported in mg CRP/mL serum.

#### 5.5.2. Nitric Oxide (NO) Assay

Nitric oxide levels in biopsy lysates and serum were determined indirectly as the total content of nitrite and nitrate (NO_3_^−^/NO_2_^−^) by spectrophotometry using the Griess method [70]. Aliquots (80 μL) of each sample (tissue samples/blood samples) were incubated with 100 μL of Griess reagent (1% sulfanilamide in 1% phosphoric acid and 0.1% naphthalene diamine dihydrochloride in water) and left at 25 °C for 10 min. Optical densities were measured spectrophotometrically (λ_540nm_) in an automatic microplate reader system ELx 800 (BioTek Instruments, Inc., Winooski, VT, USA). Nitrite concentrations in the samples were determined according to a standard curve generated by different concentrations of sodium nitrite (0.1–100 mM). Assays were performed in triplicate and NO levels were presented in μM of nitrite.

#### 5.5.3. Systemic Redox State

Changes in the systemic redox state were determined by the levels of thiobarbituric acid reactive substances (TBARS) according to the methodology described by Esterbauer and Cheeseman [71]. The production of MDA-TBA complex was determined spectrophotometrically (λ_535nm_) and presented in μM/mL of serum, using the molar extinction coefficient (ε = 1.56 × 10^5^ M^−1^cm^−1^). The levels of sulfhydryl groups (-SH) in serum were determined spectrophotometrically (λ_412nm_) by reaction with 5,5′-dithiobis-2-nitrobenzoic acid (DTNB), using the molar extinction coefficient (ε = 13600 M^−1^cm^−1^), as described by Faure and Lafond (1995) [72].

#### 5.5.4. Myeloperoxidase and N-Acethyglycosaminidase Assays

Neutrophil activation in animal inflammatory skin reaction was determined by assessing myeloperoxidase activity in skin biopsy homogenate supernatants, as previously described [73] with some modifications. Briefly, a portion of skin of animals that had undergone intradermal injection with different concentrations of crotamine were removed and frozen at −80 °C. Upon thawing, tissue specimen was homogenized (0.1 g/ mL of buffer) at 13,000 rpm by Polytron^®^ homogenizer (Kinematica, Kinematica AG, Luzern, Switzerland) in pH 4.7 buffer (NaCl 0.1 M, NaPO_4_ 0.02 M, Na-EDTA 0.015 M), centrifuged at 260× *g* for 10 min and the pellet was subjected to hypotonic lysis: 1.5 mL of 0.2% NaCl solution, followed by addition of an equal volume of a solution containing NaCl 1.6% and glucose 5%, 30 s later. After centrifugation, the pellet was resuspended in 0.05 M NaPO_4_ buffer (pH 5.4) containing 0.5% hexadecyltrimethylammonium bromide (HTAB) (Sigma-Aldrich^®^, St. Louis, MO, USA) and re-homogenized; 1-mL aliquots of the suspension were transferred to 1.5 mL polypropylene tubes followed by three freeze-thaw cycles using liquid nitrogen. Aliquots were then centrifuged for 15 min at 10,000× *g* and pellets were resuspended in buffer (0.05 mL) and assayed by measuring the change in optical density at 450 nm using tetramethylbenzidine (1.6 mM) and H_2_O_2_ (0.5 mM). Results were presented as optical density (OD) of MPO/mg tissue [73,74].

Macrophage infiltration/activation in animal inflammatory skin reaction was determined by assessing N-acetylglucosaminidase (NAG) activity in skin biopsy homogenate supernatants, according to the method described by Moscard et al. [73]. Briefly, 0.05-mL supernatant obtained from tissue homogenate was added to 30 μL p-nitrophenyl-2-acetamide-β-d-glucopyranoside (Sigma-Aldrich), diluted in 50 μL of 50 mM citrate buffer. Finally, 0.05 mL of 0.2 M glycine was added. Optical densities were measured spectrophotometrically (λ_540nm_) in an automatic microplate reader system (ELx 800, BioTeK) and results were presented as optical density (OD) of NAG/mg tissue [73].

#### 5.5.5. Histological Analysis

Animals were euthanized by deep anesthesia 1, 3 and 7 days after intradermal injections of crotamine, histamine (positive control) or sterile water (vehicle, negative control) and tissue samples from the injured dorsal skin were surgically collected. The biopsies were kept in 10% buffered formaldehyde solution, for 24 h, followed by histological processing and paraffin embedding. Sections of 5 μm were submitted to hematoxylin, eosin and Gomori trichrome staining for evaluation and quantification of inflammatory infiltrate, fibroblasts, blood vessels and collagen.

Paraffin sections of tissues were dewaxed in xylene, dehydrated in graded alcohols and washed in 0.01 M phosphate-buffered saline (PBS), pH 7.2–7.4. Endogenous peroxidase was blocked with hydrogen peroxide 0.3% in absolute methanol for 30 min. The immunolabeling procedure included negative control group injected with sterile H_2_O instead of crotamine. Primary antibody was diluted in phosphate-buffered saline (PBS) and applied overnight at 4 °C. After 2 washes in PBS, samples were incubated for 30 min at room temperature in dark chamber, and immunostained with Novolink max polymer detection system (RE7280-CE, Leica Biosystems, Wetzlar, Germany). Immunolabeling was revealed by incubation with the chromogen DAB (diaminobenzidinetetrahydrochloride) and immunohistochemical analysis was performed.

Histological sections were observed on a LEICA^®^ DM-2000 optical microscope coupled with a LEICA^®^ DFC-280 camera and LAS^®^ version 3.3.0 software (Leica Microsystem, Wetzlar, Germany). Images were captured at 200× magnification, under standardized microscopy parameters. The Plugin “Cell Counter” from ImageJ software was used for counting of blood vessels on 5 images of skin samples from each animal. In addition, the Plugin “Color Deconvolution” from ImageJ 1.48 v software (National Institutes of Health, Bethesda, MD, USA) was used to decompose the three colors of trichrome for VEGF quantification on 5 images of skin samples, as described above and the percentage of blood vessels per total area of each image was determined [73].

### 5.6. Statistical Analysis

The Kolmogorov-Smirnov and Shapiro-Wilk normality tests were run for all variables in the current study. One-way ANOVA and Tukey’s post-test were used in comparing groups. All data were presented as mean ± SD and *p* < 0.05 indicated a significant difference between groups. Graphs and statistics were performed in the GraphPad Prism 6.0 software (GraphPad Software, Inc., San Diego, CA, USA).

## Supplementary Materials

The following are available online at http://www.mdpi.com/2072-6651/11/1/39/s1, Figure S1: Increased tecidual neutrophils induced by intradermal injection of crotamine after 1, 3, and 7 days. Representative photomicrograph (HE-200 ×magnification) highlighting the inflammatory infiltrate (black arrow). The sections were stained with hematoxylin method and analyzed by light field microscopy.
