# Supplementary material for: Intradermal Application of Crotamine Induces Inflammatory and Immunological Changes In Vivo
Source: Toxins (Basel). 2019 Jan 14;11(1):39. doi: 10.3390/toxins11010039 (PMC6357061; doi:10.3390/toxins11010039)
Supplement: Supplementary file 1 [file toxins-11-00039-s001.pdf]

Supplementary Materials: Intradermal Application of Crotamine Induces Inflammatory and Immunological Changes in Vivo

Ana Vitória Pupo Silvestrini, Luana Henrique de Macedo, Thiago Antônio Moretti de Andrade, Maíra Felonato Mendes, Acácio Antônio Pigoso and Maurício Ventura Mazzi

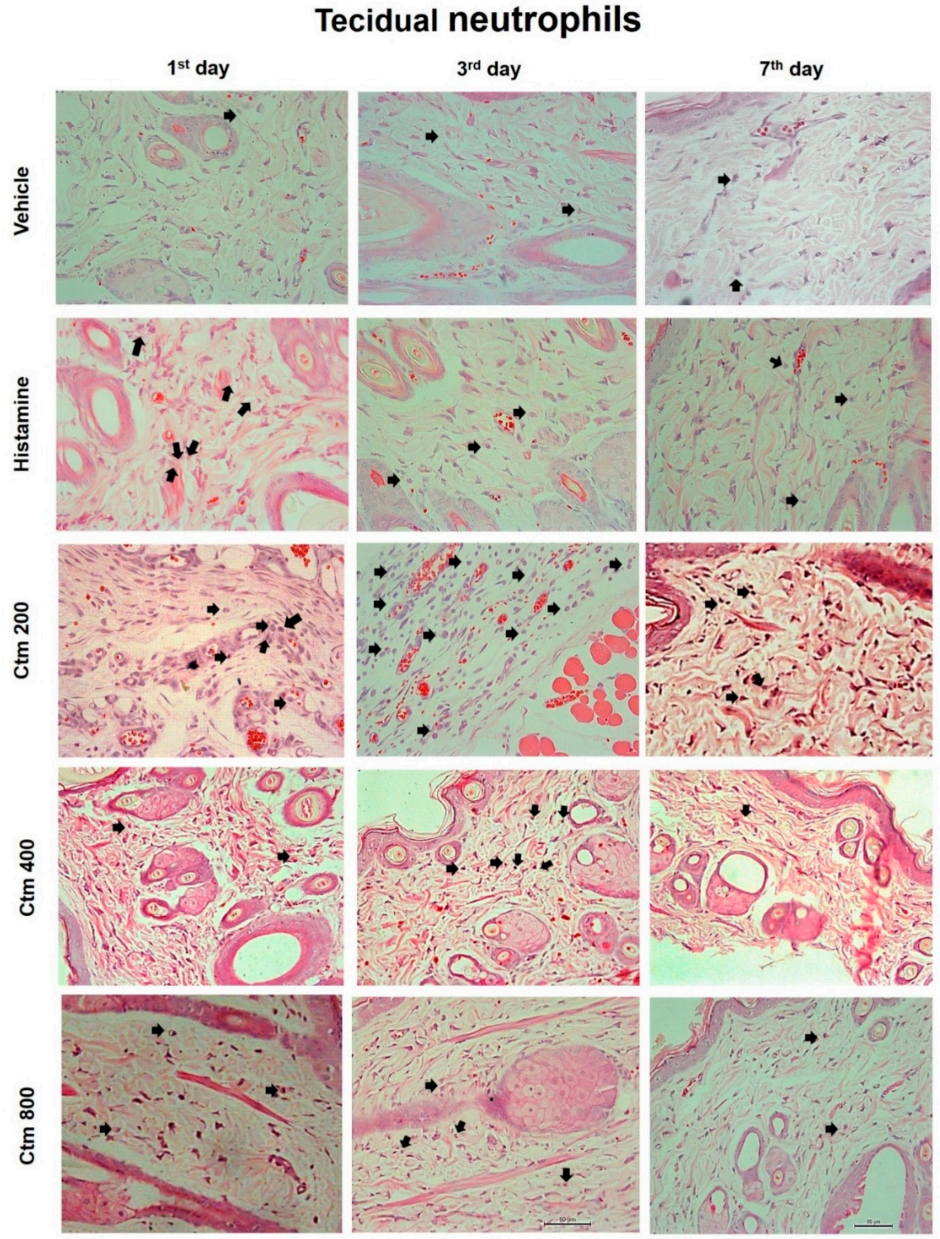

Figure S1. Increased tecidual neutrophils induced by intradermal injection of crotamine after 1, 3, and 7 days. Representative photomicrograph (HE-200 ×magnification) highlighting the inflammatory infiltrate (black arrow). The sections were stained with hematoxylin method and analyzed by light field microscopy.
